# Regioselective Separation and Extraction of Polyalkylthiophene by Metal–Organic Frameworks

**DOI:** 10.1002/anie.202513518

**Published:** 2025-08-27

**Authors:** Yu Takashima, Taku Sawayama, Nobuhiko Hosono, Takashi Uemura

**Affiliations:** ^1^ Department of Applied Chemistry Graduate School of Engineering The University of Tokyo 7‐3‐1 Hongo Bunkyo‐ku Tokyo 113‐8656 Japan; ^2^ Department of Advanced Materials Science Graduate School of Frontier Sciences The University of Tokyo 5‐1‐5 Kashiwanoha Kashiwa, Chiba 277‐8561 Japan

**Keywords:** Chromatography, Metal–organic framework, P3HT, Regioregularity, Separation

## Abstract

Here, we report a novel method for selectively extracting high‐regioregularity (RR) poly(3‐hexylthiophene) (P3HT) from crude mixtures using metal–organic frameworks (MOFs) as nanoporous separation media. This approach leverages a kinetically driven “threading” mechanism in one‐dimensional MOF channels, which discriminate subtle differences in the bonding patterns, e.g., head‐head and head‐tail linkages, in the main chain. By employing both MOF column chromatography and batch‐scale separation techniques, higher RR fractions of P3HT were readily extracted from a crude mixture containing lower RR fractions.

## Graphical abstract

We report a novel MOF‐based method for extracting high‐regioregularity poly(3‐hexylthiophene) (P3HT) from crude mixtures. Nanopore threading‐based adsorption enables recognition of erroneous head–head linkages, allowing precise isolation of regioregular P3HT. Both chromatography and batch processes demonstrate regioregularity‐dependent sorting, introducing a new paradigm in polymer purification.

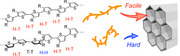

Regioregularity (RR) is among the most critical structural parameters of polymers, alongside the more commonly discussed molecular weight, molecular‐weight distribution, and tacticity. It is defined as the ratio of monomer units connected through an ideal head‐to‐tail (H─T) pattern to those through either head‐to‐head (H─H) or tail‐to‐tail (T─T) linkages. Because of the inherently stochastic nature of monomer coupling reaction, H─H or T─T linkages inevitably form during polymerization, introducing “error bonds” that disrupt the polymer's primary structure. Such erroneous connectivity can profoundly affect chain conformation and packing, thereby altering the polymer's optical properties,^[^ ^1^, ^2^ ^]^  thermal stability,^[^ ^3^, ^4^, ^5^ ^]^ and electronic behavior.^[^ ^6^, ^7^, ^8^ ^]^

Poly(3‐alkylthiophene) (P3AT), exemplified by poly(3‐hexylthiophene) (P3HT), is a representative conjugated polymer in which RR plays a pivotal role in determining electronic performance.^[^ ^8^, ^9^, ^10^, ^11^, ^12^ ^]^ Regioregular P3HT exhibits exceptionally high charge‐transport characteristics due to its regular crystal packing and well‐developed π–π stacking structures.^[^ ^9^ ^]^ However, even a slight (∼5%) reduction in RR has been reported to lower conductivity by more than an order of magnitude,^[^ ^10^ ^]^ largely because the introduction of H─H linkages induces torsion and diminishes the planarity of the conjugated polythiophene backbone.^[^ ^8^, ^12^, ^13^, ^14^, ^15^ ^]^

Over the years, substantial effort has been invested in controlled polymerization strategies aimed at achieving high RR in polymers including P3AT.^[^ ^16^, ^17^, ^18^, ^19^, ^20^, ^21^ ^]^ However, these approaches often entail complex catalyst designs and stringent reaction conditions, thus limiting their applicability to specific monomers/systems. Moreover, conventional polymer purification and separation methods, which typically rely on macroscopic characteristics of polymers (e.g., polarity, solubility, and molecular size), are not effective in identifying such subtle structural errors in the polymer backbone. Consequently, no conceptual framework has existed so far for discerning the RR of individual polymer chains and selectively isolating them from a mixture.

Here, we propose a novel approach for obtaining high‐RR P3HT by exploiting the nanopores of metal–organic frameworks (MOFs). This method enables the extraction of high‐RR fractions from mixtures that originally contain lower RR polymers with the error linkages (Figure 1). The nanopores of MOFs allow for directly accessing individual polymer chains to identify their local bonding patterns, thereby sorting P3HT by RR to achieve regioselective adsorption into the nanopores.

**Figure 1:**
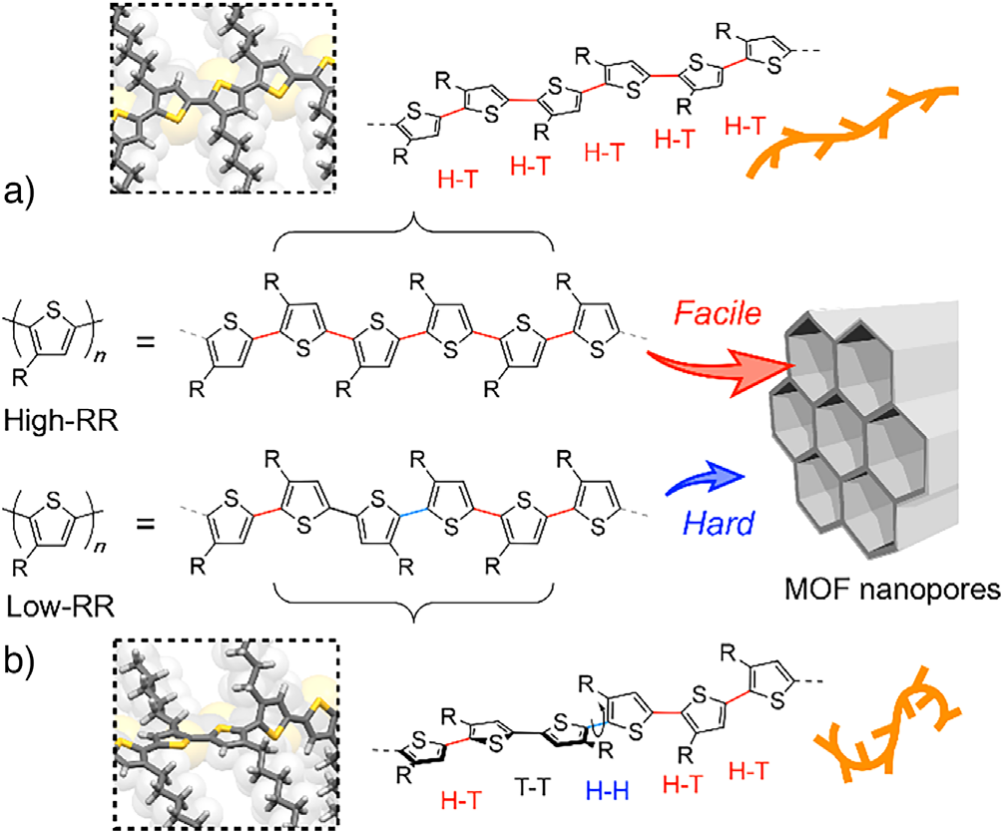
Schematic diagrams of regioselective sorting of P3HT by configured MOF nanopores. Primary structures of a) high‐RR and b) low‐RR P3HTs (R = C_6_H_13_), where H─H, H─H, and T─T linkages are color coded with red, blue, and black, respectively. High‐RR P3HT is readily inserted and adsorbed within the nanopores, whereas low‐RR P3HT with error linkages (H─H and T─T) is largely excluded. The bulkier, kinked conformation induced by torsional distortion due to the steric congestion between adjacent side chains makes nanopore insertion kinetically more difficult than in high‐RR P3HT.

MOFs offer a highly designable nanoscopic platform for molecular recognition and transformation,^[^ ^22^, ^23^, ^24^ ^]^ and have successfully addressed numerous separation challenges, particularly for small gaseous molecules.^[^ ^25^, ^26^ ^]^ Recently, our group demonstrated a technique that leverages MOF nanopores to discriminate polymer structures, allowing separation based on differences in terminal functional groups,^[^ ^27^ ^]^ topology,^[^ ^28^ ^]^ and local monomer sequences.^[^ ^29^ ^]^ Building on this approach, we have developed a MOF column chromatography method that employs MOF microcrystals as the stationary phase for polymer separations.^[^ ^30^, ^31^, ^32^, ^33^, ^34^ ^]^ In the present work, exploiting the regioselective adsorption mechanism coupled with MOF column chromatography technique, we successfully and readily extracted high RR fractions from a conventionally polymerized lower RR crude mixture of P3HT.

To evaluate the fundamental RR‐recognition capability of MOFs, we first examined discrete hexylthiophene dimers (**D**) bearing deliberately introduced H─T, H─H, and T─T linkages, namely **D_H‐T_**, **D_H‐H_**, and **D_T‐T_**, respectively, via MOF column chromatography (Figure 2). For the stationary phase, we selected [Zn_2_(ndc)_2_(ted)]*_n_* (hereafter **1**; ndc = 1,4‐naphthalenedicarboxylate, ted = triethylenediamine),^[^ ^35^ ^]^ [Zn_2_(bdc)_2_(ted)]*_n_* (**2**; bdc = 1,4‐benzenedicarboxylate),^[^ ^36^ ^]^ and [In(bdc)(OH)]*_n_* (**3**) (Figures S1–S3).^[^ ^37^, ^38^ ^]^ These MOFs feature one‐dimensional (1D) channels along the *c*‐axis, with pore diameters of 0.57 nm (**1**), 0.75 nm (**2**), and 1.7 nm (**3**), respectively. Each MOF was synthesized via solvothermal reactions under stirring, and the resulting powdery product was packed into stainless‐steel columns (4 mm I.D. × 50 mm L.) to form Columns **1**, **2**, and **3** (see SI).^[^ ^27^, ^32^ ^]^  The particle size of each MOF filled in the column was 10.0 ± 4.9 µm (**1**), 9.2 ± 2.6 µm (**2**), and 8.6 ± 4.4 µm (**3**), respectively (Figures S4 and S5). The dimeric regioisomers were subjected to high‐performance liquid chromatography (HPLC) on each MOF column, using *n*‐hexane as the eluent (see Supporting Information).

**Figure 2:**
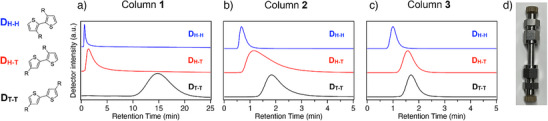
Chemical structures and HPLC chromatograms of **D_H‐H_** (blue), **D_H‐T_** (red), and **D_T‐T_** (black), recorded on a) Column **1**, b) Column **2**, and e) Column **3** (eluent: *n*‐hexane, temperature: 40 °C, flow rate: 1.0 mL min^−1^). d) A photograph of a representative MOF‐packed column used for the HPLC experiments (4 mm I.D. × 50 mm L.).

Interestingly, these MOF columns distinguished the dimeric isomers by exhibiting retention times in the order of **D_H‐H_** < **D_H‐T_** < **D_T‐T_** (Figure 2). **D_H‐H_** adopts a nonplanar, twisted conformation due to the steric hindrance of its hexyl side chains, as evidenced by its UV–vis absorption spectrum in *n*‐hexane solution (Figures S6 and S7). This twisted structure likely prevents **D_H‐H_** from entering the pores, resulting in weaker adsorption affinity and consequently the shortest retention times on all MOF columns. In contrast, both **D_H‐T_** and **D_T‐T_** exhibit significant retention, with **D_T‐T_** in particular having the longest retention time (Figure 2). This retention behavior can be attributed to the nanopore insertion mechanism: **D_T‐T_** can adopt a straight conformation by extending both hexyl chains at both ends (Figure S6), which facilitates insertion and leads to stronger adsorption affinity compared to other isomers unable to adopt such an extended shape. Similar shape‐based discrimination via MOF nanopores has been demonstrated by our group for other systems, including polymers, where a bulkier or kinked region of the chain is excluded from the pores, thereby allowing the detection of subtle structural differences.^[^ ^27^, ^34^ ^]^ Notably, selectivity for **D_T‐T_** increases as pore size decreases, reaching its highest on Column **1** (Figure 2a).

Additionally, we observed that the choice of eluent significantly impacts retention behavior. While all **D** isomers achieved good resolution on all MOF columns under *n*‐hexane eluent, no retention was observed when chloroform was used. Based on our previous work on polymer adsorption in MOF nanopores, this result can be attributed to the balance between the analyte's solubility in the solvent and its affinity for the MOF stationary phase, in addition to other possible causes such as polarity effect and electrostatic interactions.^[^ ^32^, ^33^ ^]^ In chloroform, where alkylated poly‐ and oligothiophene derivatives are generally highly soluble,^[^ ^39^ ^]^ the **D** isomers predominantly remain in the mobile phase, weakening adsorption. Conversely, *n*‐hexane, a relatively poor solvent for these materials, enhances their adsorption into the nanopores. Consequently, by employing mixtures of hexane and chloroform with varying ratios, we can fine‐tune the retention behavior (Figure S8).

Building on the intriguing ability of the MOFs to discriminate **D** isomers, we hypothesized that this nanopore insertion‐based approach could also enable the recognition of RR of their polymeric version, P3HT. In P3HT chains, erroneous H─H linkages entail steric hindrance between side chains, leading to local torsion and a kinked, non‐linear backbone conformation (Figure 1).^[^ ^40^ ^]^  In contrast, H─T and T─T linkages are less sterically hindered and promote a more linear chain conformation. Therefore, the ability to recognize and exclude H─H linkages at the single‐chain level is key to achieving RR‐selective polymer sorting. In this context, we opted for **3** as the most promising candidate, as it demonstrated the highest resolution in separating the **D_H‐H_** isomer from **D_H‐T_** and **D_T‐T_** in the dimer studies (Figure 2c). We thus anticipated that **3** could similarly extract high‐RR P3HT fractions from crude mixtures by selectively adsorbing chains with more linear, H─H‐free backbones.

Before the chromatographic study, we investigated the direct insertion of P3HT into bulk **3** to examine whether regioselective extraction of P3HT is feasible. The P3HT sample, prepared via the Grignard metathesis (GRIM) method (see Supporting Information),^[^ ^20^ ^]^ possessed a relatively high RR of 90.2% (hereafter denoted as **P_90_**; *M*_n_ = 15,700 relative to polystyrene standards), as determined by ^1^H NMR based on the integration ratio of H─T (2.84–2.76 ppm) and H─H (2.62–2.50 ppm) signals (Table S1).^[^ ^41^ ^]^ In this study, we chose the GRIM method to obtain relatively high‐RR P3HT as the crude material instead of the more common oxidative polymerization, which typically yields lower‐RR (regiorandom) P3HT. This approach was taken because regiorandom samples likely contain very few high‐RR chains, reflecting the binomial distribution inherent to their statistical monomer‐coupling process (Figure S9). We employed a solvent‐assisted insertion method previously developed in our group: powdered **3** (120 mg) was immersed in a chloroform solution of **P_90_** (40 mg), allowed to stand at 25 °C during slow solvent evaporation, and subsequently dried under vacuum.^[^ ^31^ ^]^  The resulting solid was washed with a 7:3 (v/v) chloroform/hexane mixture to remove residual polymers not incorporated into the MOF, affording an inclusion complex (**3**⊃P3HT) (see Supporting Information). A portion of the inclusion complex was digested with an EDTA·4Na solution, then analyzed by ^1^H NMR, revealing that 12 mg/g of P3HT was loaded in **3** (Table S2).

Powder X‐ray diffraction (PXRD) revealed no significant change in the crystallinity of **3** (Figure S2), and scanning electron microscope (SEM) images confirmed that the rod‐like morphology of **3** was retained upon P3HT insertion (Figure S4). N_2_ adsorption measurements of the composite showed a reduction in adsorption capacity and effective pore volume, indicative of successful P3HT insertion into the nanopores (Figure S10). Interestingly, the obtained composite, **3**⊃P3HT, appeared reddish in color (Figure 3a). Its UV–vis spectrum exhibited multiple absorption bands centered around 540 nm, closely resembling those of a bulk P3HT film, where P3HT chains adopt a planar, extended conformation with well‐developed interchain π‐π stacking (Figure 3b).^[^ ^10^, ^42^ ^]^ On the other hand, its fluorescence spectrum exhibited a unique shoulder peak around 580 nm that was absent in the bulk P3HT film, indicative of confinement of a few chains within the nanochannel (Figure 3c).^[^ ^43^ ^]^  These findings are consistent with our previous studies, confirming the successful inclusion of P3HT chains in the 1D channels of **3**.

**Figure 3:**
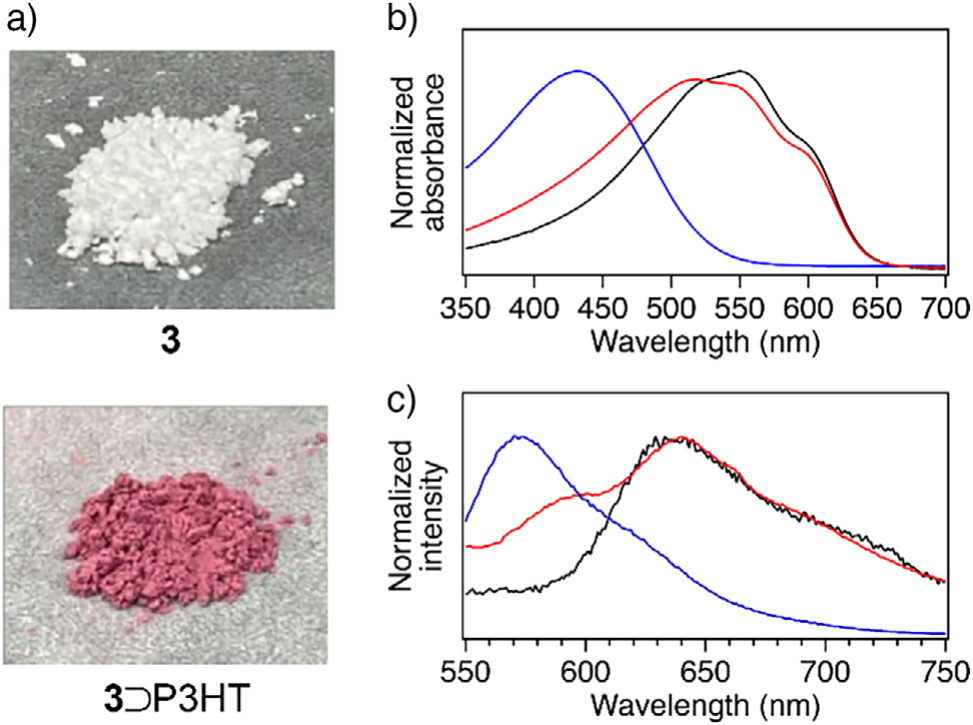
a) Photographs of pristine **3** (top) and **3**⊃P3HT composite (bottom). b) UV–vis absorption spectra and c) fluorescence spectra for a chloroform solution (blue) and a thin film (black) of pristine P3HT (**P_90_**), and **3**⊃P3HT composite (red), measured at 25 °C.

After forming the composite, we recovered the inserted P3HT by digesting the host **3** framework (see Supporting Information). Interestingly, ^1^H NMR analysis of the extracted P3HT revealed the RR value of 94.2% (Table S2). The 4% increase of RR from the initial 90.2%‐RR is significant given that, according to the binomial distribution typical of statistical monomer coupling, P3HT chains above 94% RR constitute only ∼13% of the total population in the initial sample (Figure S9). These results confirm that **3** discriminates the local bonding patterns of individual P3HT chains upon insertion, preferentially accommodating higher‐RR species. Noted that the extracted P3HT had a molecular weight of *M*_n_ = 12,600 g mol^−1^, which is slightly smaller than the initial crude mixture. This is attributed to a kinetic effect, as shorter chains generally diffuse more quickly within the pore.^[^ ^33^ ^]^

We found that temperature is crucial in determining the efficiency of this regioselective polymer sorting. Repeating the experiment using a higher insertion temperature of 70 °C yielded a lower RR increment (reaching 92.1%) but a higher loading of 90 mg/g (Table S2). Likewise, the initial RR of the crude P3HT influenced the outcome: using P3HT with 93.7% RR (**P_94_**) led to a loading of 38 mg/g and a final RR of 97.5% through the insertion step at 25 °C (Table S2). It should be noted that no dependence of RR on molecular weight was observed (Figure S11). These observations align with our proposed, kinetically driven recognition mechanism, as previously inferred from dimer separation studies. In solution, low‐RR P3HT adopts a winding, disordered conformation with reduced persistence length compared to high‐RR analogue due to the presence of H─H linkages in the main chain.^[^ ^40^ ^]^ Accordingly, such chains must overcome an additional energy barrier to straighten before entering the pores, whereas high‐RR chains, already more linear, intercalate readily, resulting in kinetically favored adsorption (Figure 1).

Encouraged by the promising regioselective sorting capability of **3**, we next examined the separation of P3HT on HPLC. Two authentic P3HT samples, one with high RR (96.6%, denoted as **P_97_**) and the other with low regioregularity (76.8%, **P_77_**), were employed (Table S1). Both samples were synthesized separately and fractionated to achieve identical molecular weights with narrow distribution (*M*_n_ ∼21,000, *Ð* ∼1.1, Table S1, and Figure S12) so that any differences in LC retention would reflect their RR rather than chain length. Despite their close structural similarities, UV–vis absorption spectra showed a longer maximum absorption wavelength for **P_97_** (*λ*_max_ = 449 nm), indicating extended conjugation relative to **P_77_** (*λ*_max_ = 434 nm) owing to the higher linearity of chain (Figure S13).^[^ ^40^ ^]^

We analyzed both **P_97_** and **P_77_** on Column **3** using HPLC with a solvent gradient of hexane/chloroform to modulate adsorption strength. We employed a relatively slow flow rate of 0.1 mL min^−1^ to maximize separation efficiency, taking into account the kinetically driven recognition mechanism.^[^ ^34^ ^]^  By starting with a 30% *n*‐hexane eluent (*n*‐hexane/chloroform = 30/70, v/v) and gradually shifting to 100% chloroform, higher‐RR P3HT was expected to remain adsorbed longer (see Supporting Information). Indeed, **P_97_** eluted later (retention time, *t* = 6.30 min, 1.26 CV) than **P_77_** (*t* = 6.07 min, 1.21 CV), confirming that higher RR correlates with stronger adsorption under these conditions (Figure 4a). To further validate this separation capability, we also analyzed a binary mixture of **P_97_** and **P_77_** (1:1, w/w) under the same conditions. Although peak broadening prevented clear resolution of the two fractions, time‐resolved UV–vis spectra (acquired with an in‐line photodiode array detector) confirmed a progressive red shift of maximum absorption wavelength (*λ*_max_) during elution (Figure 4b,c), indicative of successful RR‐based separation in the column. Specifically, the lower‐RR fraction eluted first, followed by the higher‐RR fraction. These results on HPLC demonstrate that Column **3** can effectively separate not only smaller oligomers but also full‐length polymers based on a regioselective adsorption mechanism. It should be noted that Columns **1** and **2** showed no separation for **P_97_** and **P_77_** under identical conditions (Figure S14), likely due to their narrower pore sizes, which limit polymer adsorption as a result of kinetic constraints.^[^ ^32^ ^]^

**Figure 4:**
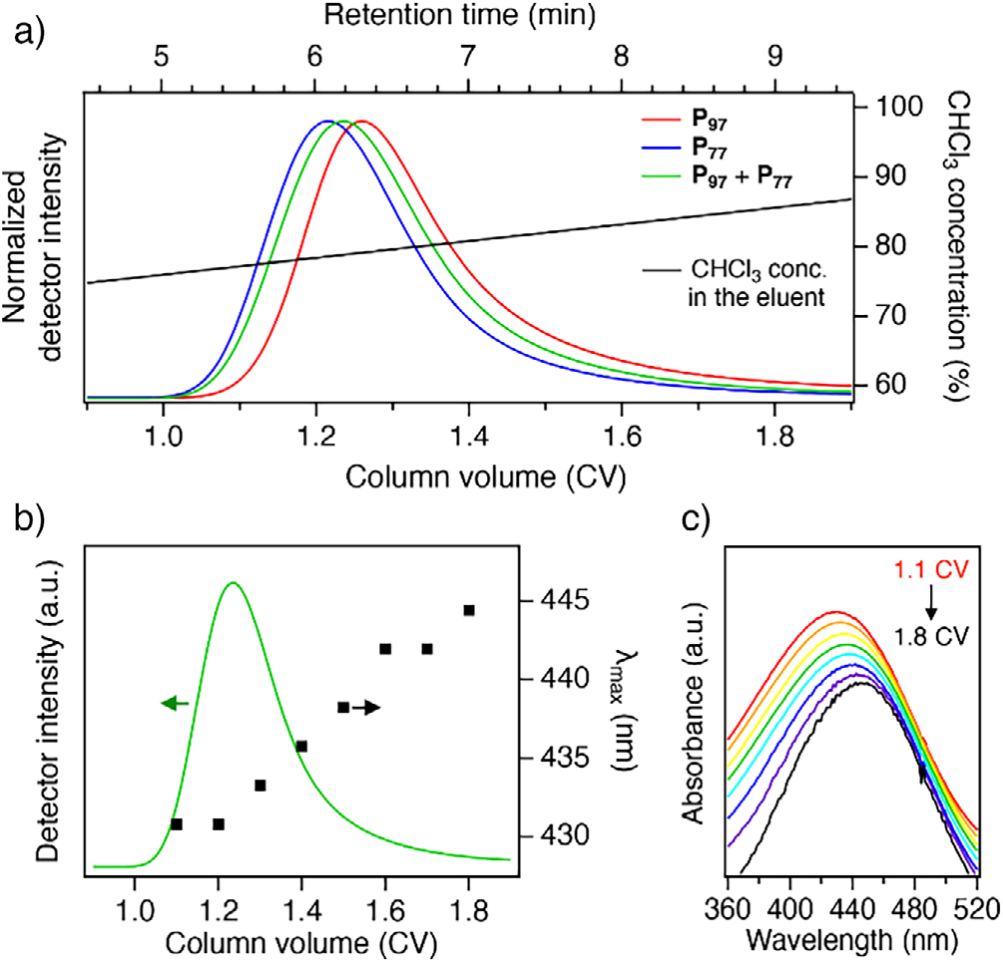
a) HPLC chromatograms of **P_97_** (red), **P_77_** (blue), and the mixture of **P_97_**/**P_77_** (1/1, w/w) (gradient: hexane/chloroform = 30/70 (0.5 CV) | 30/70‐(2.5 CV)‐0/100; temperature: 30 °C; flow rate: 0.1 mL min^−1^; monitoring wavelength = 433 nm). b) The HPLC chromatogram of the **P_97_**/**P_77_** mixture (1/1, w/w), plotted with *λ*_max_ observed in the UV–vis spectra recorded at the respective retention time (right axis). c) The UV–vis spectra recorded at the retention times of 5.5, 6.0, 6.5, 7.0, 7.5, 8.0, 8.5, and 9.0 min (1.1–1.8 CV).

Finally, we performed fractionation of high‐RR P3HT from a low‐RR mixture using a conventional medium‐pressure flash chromatography apparatus (Isolera One, Biotage). A preparative column (20 mm I.D. × 150 mm L.) packed with approximately 13 g of **3** was prepared (Figure 5a, see Supporting Information). We then loaded 20 mg of **P_90_** onto the column and eluted with a hexane/chloroform gradient (30/70 to 0/100, v/v) over 1.33 CVs (see Supporting Information). Monitoring at 400 nm on an UV detector revealed an elution peak from 1.0 to 3.0 CV (Figure 5b). The eluted P3HT was fractionated at different CVs. After the solvent had evaporated, each P3HT fraction was analyzed by ^1^H NMR to determine its RR. Plotting the RR values against elution time (i.e., CV) revealed a progressive increase from 89% to over 95% (Figure 5b), thereby confirming the regioselective separation of P3HT. The P3HT collected in the 95%‐RR fraction was only 0.04 mg (0.2% of the original **P_90_** amount), likely reflecting the low abundance of higher‐RR chains in **P_90_** (Figure S9) and too strong adsorption of extremely high‐RR chains in the column. Indeed, the overall recovery was 66%, and the column exhibited a slight color change (white to light purple) after use (Figures S15 and S16), implying the possible permanent adsorption. We anticipate that this issue could be mitigated by employing temperature‐controlled preparative LC systems.

**Figure 5:**
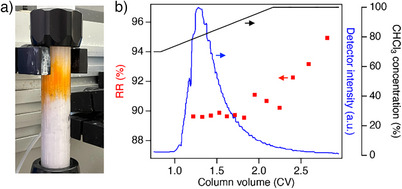
a) A photograph of the preparative column of **3** (20 mm I.D. × 150 mm L.), in operation on a middle‐pressure flash chromatography apparatus. b) The chromatogram for the flash chromatography of **P_90_** using the preparative column of **3**, plotted with the RR value of each fraction collected within the given retention time frame. (eluent: hexane/chloroform = 30/70 (0.8 CV) | 30/70‐(1.33 CV)‐0/100, v/v; temperature: 25 °C; flow rate: 5.0 mL min^−1^; monitoring wavelength = 400 nm).

In conclusion, our results demonstrate that MOFs can discriminate P3HT chain bonding patterns and sort them based on RR, enabling the enrichment of high‐RR P3HT from a lower‐RR crude mixture. The MOF column chromatography technique facilitates both the separation of oligothiophene isomers and the regioselective extraction of P3HT using standard LC setups. Additionally, batch‐scale adsorptive separation proved equally effective at extracting higher‐RR P3HT. We attribute this robust RR‐recognition capability to a nanopore‐based, kinetically driven threading mechanism. We anticipate that such regioselective extraction via nanopores will provide a new paradigm in the production of regioregular polymers, wherein intricate synthetic routes have traditionally been considered indispensable.

## Supporting Information

The authors have cited additional references within the Supporting Information.^[44–46]^

## Conflict of Interests

The authors declare no conflict of interest.

## Supporting information



Supplementary Information

## Data Availability

The data that support the findings of this study are available in the supplementary material of this article.
