# Supplementary material for: Regioselective Separation and Extraction of Polyalkylthiophene by Metal–Organic Frameworks
Source: Angew Chem Int Ed Engl. 2025 Aug 27;64(41):e202513518. doi: 10.1002/anie.202513518 (PMC12501673; doi:10.1002/anie.202513518)
Supplement: Supplementary file 1 — Supplementary Information [file ANIE-64-e202513518-s001.pdf]

Supporting Information for

Regioselective Separation and Extraction of Polyalkylthiophene by Metal–Organic Frameworks

Yu Takashima, Taku Sawayama, Nobuhiko Hosono,* Takashi Uemura*

Table of Contents

1. General Instruments..........2
2. Materials..........2
3. Synthesis..........3
4. HPLC on MOF Columns..........6
5. Batch Adsorption and Extraction Procedures..........7
6. Flash Chromatography on a preparative MOF Column..........7
7. Supporting Tables..........8
8. Supporting Figures..........9
9. Supporting References..........25

1. General Instruments

^{1}H nuclear magnetic resonance (NMR) spectra were recorded using Bruker Avance III HD (500 MHz) spectrometers equipped with a PABBO probe. Scanning electron microscopy (SEM) was performed using Hitachi S3000N at an accelerating voltage of 5 kV. The samples were deposited on conducting carbon tape attached to the sample stage and then sputter-coated with osmium. Powder X-ray diffraction (PXRD) data were recorded using a Rigaku SmartLab X-ray diffractometer with Cu Kα radiation. Nitrogen gas adsorption measurements were performed using the volumetric method with a MicrotracBEL BELSORP mini and BELSORP MAX X. The sample was dried and evacuated at 150 °C for 16 h under vacuum before measurement. Preparative recycling size-exclusion chromatography (SEC) was carried out using Japan Analytical Industry LaboACE-5060 equipped with JAIGEL-2HR column. The mobile phase was chloroform at a flow rate of 10.0 mL/min. Analytical SEC was performed at 40 °C using Shimadzu LC-2050C equipped with two polystyrene gel columns in series (Shodex LF-804). The mobile phase was tetrahydrofuran (THF) at a flow rate of 1.0 mL/min. The molecular weights and polydispersity index ($Đ$ = M_w/M_n) were obtained relative to a polystyrene standard (SM-105, Shodex). UV–vis spectra were recorded on a JASCO model V-670 spectrometer. Fluorescence spectra were measured by using HORIBA Jobin Yvon Fluorolog-3 spectrophotometer with double-grating monochromators, a 450 W xenon lamp, and a R928 photomultiplier tube run in photon-counting mode. Particle size distribution analysis was carried out using HORIBA Partica LA-950. The samples were dispersed in acetone at 25 °C. Preparative middle-pressure flash chromatography was performed at 25 °C using Biotage Isorera One.

2. Materials

All reagents and chemicals used in this study were obtained from FUJIFILM Wako Pure Chemicals and TCI Chemicals, unless otherwise noted.

3. Synthesis

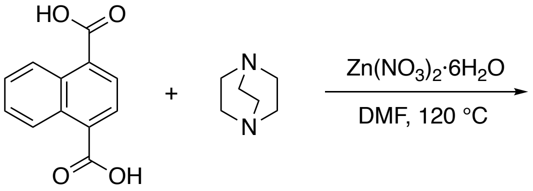

1

Synthesis of 1. $[Zn_2(ndc)_2(ted)]_n$ (**1**) was synthesized following a procedure adapted from the literature.[35] The solvothermal synthesis of **1** was performed with stirring the reaction mixture to have a better control of particle size. A *N*,*N*-dimethylformamide (DMF) (30 mL) solution of $Zn(NO_3)_2{\cdot}6H_2O$ (3.41 g, 11.5 mmol) was added a DMF solution (30 mL) of 1,4-naphthalenedicarboxylic acid (2.48 g, 11.5 mmol) and triethylenediamine (0.643 g, 5.74 mmol). The mixture was stirred at 120 °C for 48 h. The reaction mixture was then cooled to 25 °C and the powdery solid was collected by suction filtration. The product was washed with anhydrous DMF (ca. 100 mL) on the funnel to give **1** (5.25 g) with DMF as a crystal solvent, which was activated in vacuo at 120 °C prior to use. The experimental PXRD pattern closely matched the simulated pattern, confirming the successful synthesis of **1** (Figure S2).[35] Particle size: 10.0 ± 4.9 µm (Figures S4 and S5).

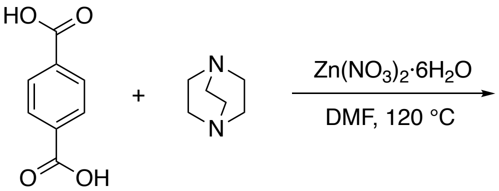

2

Synthesis of 2. $[Zn_2(bdc)_2(ted)]_n$ (**2**) was synthesized following a procedure adapted from the literature.[36] The solvothermal synthesis of **2** was performed with stirring the reaction mixture to have a better control of particle size. A DMF (30 mL) solution of $Zn(NO_3)_2{\cdot}6H_2O$ (3.41 g, 11.5 mmol) was added a DMF solution (30 mL) of 1,4-benzenedicarboxylic acid (1.91 g, 11.5 mmol) and triethylenediamine (0.643 g, 5.74 mmol). The mixture was stirred at 120 °C for 48 h. The reaction mixture was then cooled to 25 °C and the powdery solid was collected by suction filtration. The product was washed with anhydrous DMF (ca. 100 mL) on the funnel to give **2** (2.91 g) with DMF as a crystal solvent, which was activated in vacuo at 120 °C prior to use. The experimental PXRD pattern closely matched the simulated pattern, confirming the successful synthesis of **2** (Figure S2).[36] Particle size: 9.2 ± 2.6 µm (Figures S4 and S5).

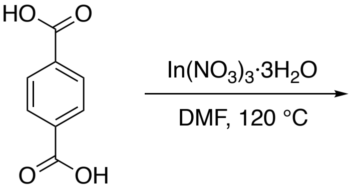

3

Synthesis of 3. $[In(bdc)(OH)]_n$ (**3**) was synthesized following a procedure adapted from the literature.[38] $In(NO_3)_3{\cdot}3H_2O$ (25.2 g, 71.0 mmol) and 1,4-benzenedicarboxylic acid (11.8 g, 71.0 mmol) were added

to DMF (400 mL), and the mixture was stirred at 25 °C until the solution became clear. The mixture was stirred at 120 °C for 24 h. The reaction mixture was then cooled to 25 °C and the product was collected by suction filtration. The product was washed with methanol (ca. 100 mL) on the funnel. Residual solvent was removed under reduced pressure at 150 °C overnight to give **3** as white powder (24.9 g). The experimental PXRD pattern closely matched the simulated pattern, confirming the successful synthesis of **3** (Figure S2).[38] Particle size: 8.6 ± 4.4 µm (Figure S4 and S5).

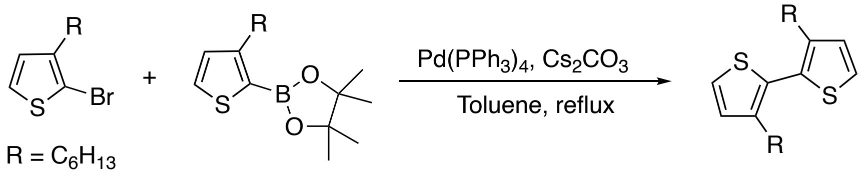

Synthesis of $D_{H\text{-}H}$. 3,3'-dihexyl-2,2'-bithiophene (**$D_{H\text{-}H}$**) was synthesized following a procedure adapted from the literature.[44] 2-bromo-3-hexylthiophene (0.84 g, 3.4 mmol), 2-(3-hexylthiophen-2-yl)-4,4,5,5-tetramethyl-1,3,2-dioxaborolane (1.00 g, 3.4 mmol), and Cs_2CO_3 (2.7 g, 8.5 mmol) were placed in a Schlenk flask with toluene (30 mL). The solution was degassed by bubbling nitrogen for 10 min. $Pd(PPh_3)_4$ (0.225 g, 0.194 mmol) was added, and the solution was refluxed overnight under inert atmosphere. The solution was cooled to 25 °C and then diluted with *n*-hexane, filtered and concentrated under reduced pressure. The resulting crude product was purified through flash chromatography (SiO_2, *n*-hexane), followed by preparative recycling SEC to give **$D_{H\text{-}H}$** (0.17 g, 75% yield) as a colorless oil. ^1H NMR (500 MHz, $CDCl_3$): 7.28 (2H, d, J = 5.2 Hz), 6.96 (2H, d, J = 5.2 Hz), 2.49 (4H, t, J = 7.8 Hz), 1.49-1.56 (4H, m), 1.18-1.29 (12H, m), 0.85 (6H, t, J = 7.0 Hz).

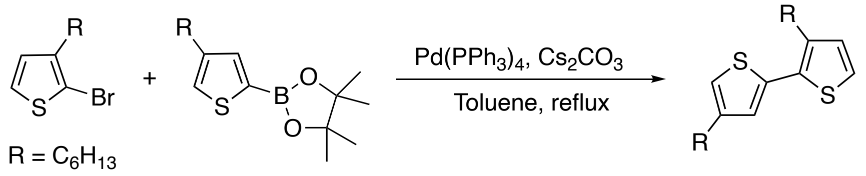

Synthesis of $D_{H\text{-}T}$. 3,4'-dihexyl-2,2'-bithiophene (**$D_{H\text{-}T}$**) was synthesized following a procedure adapted from the literature.[45] 2-bromo-3-hexylthiophene (0.22 g, 0.90 mmol), 2-(4-hexylthiophen-2-yl)-4,4,5,5-tetramethyl-1,3,2-dioxaborolane (0.26 g, 0.90 mmol), and Cs_2CO_3 (0.73 g, 2.2 mmol) were placed in a Schlenk flask with toluene (15 mL). The solution was degassed by bubbling nitrogen for 10 min. $Pd(PPh_3)_4$ (0.052 g, 0.045 mmol) was added, and the solution was refluxed overnight under inert atmosphere. The solution was cooled to 25 °C and then diluted with *n*-hexane, filtered and concentrated under reduced pressure. The resulting crude product was purified through flash chromatography (SiO_2, *n*-hexane), followed by preparative recycling SEC to give **$D_{H\text{-}T}$** (0.24 g, 81% yield) as a colorless oil. ^1H NMR (500 MHz, $CDCl_3$): 7.14 (1H, d, J = 5.4 Hz), 6.93 (d, 1H, J = 1.4), 6.91 (1H, d, J = 5.2 Hz), 6.88 (1H, m), 2.74 (2H, t, J = 7.9 Hz), 2.60 (2H, t, J = 7.7 Hz), 1.58-1.67 (4H, m), 1.23-1.39 (12H, m), 0.86-0.91 (6H, m).

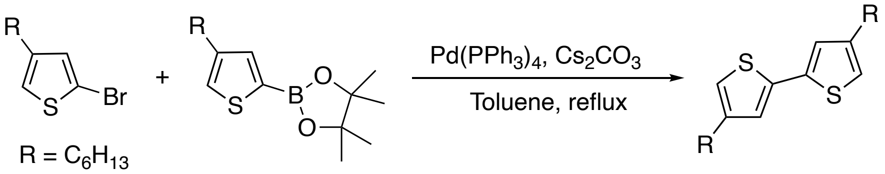

Synthesis of $D_{T\text{-}T}$. 4,4'-dihexyl-2,2'-bithiophene (**$D_{T\text{-}T}$**) was synthesized following a procedure adapted from the literature.[45] 2-bromo-4-hexylthiophene (0.22 g, 0.90 mmol), 2-(4-hexylthiophen-2-yl)-4,4,5,5-tetramethyl-1,3,2-dioxaborolane (0.26 g, 0.90 mmol), and Cs_2CO_3 (0.73 g, 2.2 mmol) were placed in a Schlenk flask with toluene (15 mL). The solution was degassed by bubbling nitrogen for 10 min. $Pd(PPh_3)_4$ (0.059 g, 0.051 mmol) was added, and the solution was refluxed overnight under inert atmosphere. The solution was cooled to 25 °C and then diluted with *n*-hexane, filtered and concentrated under reduced pressure. The resulting crude product was purified through flash chromatography (SiO_2, *n*-hexane), followed by preparative recycling SEC to give **$D_{T\text{-}T}$** (0.62 g, 69% yield) as a colorless oil. ^{1}H NMR (500 MHz, $CDCl_3$): 6.98 (2H, s), 6.77 (2H, s), 2.57 (4H, t, J = 7.7 Hz), 1.58-1.66 (4H, m), 1.25-1.39 (12H, m), 0.90 (6H, t, J = 6.8 Hz).

Synthesis of P_{90}. P3HT with 90.2% RR (**P_{90}**) was synthesized using the GRIM method, following a procedure adapted from the literature.[20] 2,5-Dibromo-3-hexylthiophene (3.0 g, 9.2 mmol) was dissolved in anhydrous THF (90 mL). Methylmagnesium bromide (7.4 mL of 1.0 M THF solution) was added to the solution, and the mixture was heated to reflux for 1 h. Then, dichloro[1,3-bis(diphenylphosphino)propane]nickel (Ni(dppp)Cl_2) (0.039 g, 0.073 mmol) was added to the mixture, and the solution was stirred at reflux for 2 h. The mixture was cooled to 25 °C, poured into 150 mL of methanol, and then filtered into a Soxhlet thimble. Soxhlet extractions were performed with methanol (to remove monomer and salts), hexanes (to remove catalyst and oligomers), and chloroform for 24 h each. The chloroform fraction was collected and evaporated to dryness under reduced pressure, affording P3HT (0.30 g) (**P_{90}**). ^{1}H NMR (500 MHz, $CDCl_3$): 6.98, 2.80, 2.56, 1.66-1.74, 1.30-1.48, 0.91. RR: 90.2%. M_n: 15,700, $Đ$: 1.36 (Table S1).

Synthesis of P_{94}. P3HT with 93.7% RR (**P_{94}**) was synthesized using the GRIM method, following a procedure adapted from the literature.[20] 2,5-Dibromo-3-hexylthiophene (1.3 g, 4.0 mmol) was dissolved in anhydrous THF (18 mL). Methylmagnesium bromide (3 mL of 1.0 M THF solution) was added to the solution, and the mixture was heated to reflux for 1 h. Then, dichloro[1,3-bis(diphenylphosphino)propane]nickel (Ni(dppp)Cl_2) (0.017 g, 0.031 mmol) was added to the mixture, and the solution was stirred at reflux for 2 h. The mixture was cooled to 25 °C, poured into 150 mL of methanol, and then filtered into a Soxhlet thimble. Soxhlet extractions were performed with methanol (to remove monomer and salts), hexanes (to remove catalyst and oligomers), and chloroform for 24 h each. The chloroform fraction was collected and evaporated to dryness under reduced pressure, affording P3HT (0.24 g) (**P_{94}**). ^{1}H NMR (500 MHz, $CDCl_3$): 6.98, 2.80, 2.56, 1.66-1.74, 1.30-1.48, 0.91. RR: 93.7%. M_n: 22,400, $Đ$: 1.41 (Table S1).

Synthesis of P_{97}. P3HT with 96.6% RR (**P_{97}**) was synthesized using the GRIM method, following a procedure adapted from the literature.[20] 2,5-Dibromo-3-hexylthiophene (8.0 g, 24.5 mmol) was

dissolved in anhydrous THF (100 mL). Methylmagnesium bromide (20 mL of 1.0 M THF solution) was added to the solution, and the mixture was heated to reflux for 1 h. Then, dichloro[1,3-bis(diphenylphosphino)propane]nickel (Ni(dppp)Cl_2) (0.106 g, 0.196 mmol) was added to the mixture, and the solution was stirred at reflux for 2 h. The mixture was cooled to 25 °C, poured into 150 mL of methanol, and then filtered into a Soxhlet thimble. Soxhlet extractions were performed with methanol (to remove monomer and salts), hexanes (to remove catalyst and oligomers), and chloroform for 24 h each. The chloroform fraction was collected and evaporated to dryness under reduced pressure, affording P3HT (0.24 g) ($\mathbf{P_{97}}$). ^{1}H NMR (500 MHz, $CDCl_3$): 6.98, 2.80, 2.56, 1.66-1.74, 1.30-1.48, 0.91. RR: 96.6%. M_n: 21,200, $Đ$: 1.07 (Table S1).

Synthesis of $\mathbf{P_{77}}$. P3HT with 76.8% RR ($\mathbf{P_{77}}$) was synthesized using the oxidative polymerization method, following a procedure adapted from the literature.[41] $FeCl_3$ (1.9 g, 12 mmol) and chloroform (15 mL) were placed in a two-necked flask. The flask was then purged with nitrogen, and 3-hexylthiophene (0.50 g, 3.0 mmol) was added via a syringe to the suspension. The resulting black mixture was stirred for 24 h under the nitrogen atmosphere at 25 °C. The reaction mixture was poured into 150 mL of methanolic HCl (10 wt%). The precipitate was collected by filtration and washed with methanol. Soxhlet extractions were performed with methanol and chloroform for 24 h each. The chloroform fraction was collected and evaporated to dryness under reduced pressure, affording low-RR P3HT (0.22 g). The resultant P3HT was then fractionated using preparative recycling SEC into different molecular weight fractions. The fractions containing P3HT with molecular weight close to that of $\mathbf{P_{97}}$ (M_n = 21,000) were combined to obtain $\mathbf{P_{77}}$. ^{1}H NMR (500 MHz, $CDCl_3$): 6.94-7.06, 2.80, 2.56, 1.66-1.74, 1.30-1.48, 0.91. RR: 76.8%. M_n: 21,000, $Đ$: 1.08 (Table S1).

4. HPLC on MOF Columns

The MOF-packed HPLC columns were prepared using a tapping method adapted from the literature.[27,28] Specifically, 0.3–0.4 g of dried **1**, **2**, or **3** was placed into a stainless-steel column (4 mm I.D. × 50 mm L.; GL Sciences 6010-11041) and packed by tapping, resulting in Columns **1**, **2**, and **3**, respectively.

The MOF-packed column, Column **1**, **2**, and **3**, was connected to a Shimadzu model HPLC Prominence equipped with a refractive index detector (Shimadzu RID-20A) and a photodiode array (PDA) detector (Shimadzu SPD-M20A).

For the HPLC analysis of $\mathbf{D_{H\text{-}H}}$, $\mathbf{D_{H\text{-}T}}$, and $\mathbf{D_{T\text{-}T}}$ on the MOF columns, isocratic elution using *n*-hexane (100%) was employed (flow rate: 1 mL/min). The column temperature was 40 °C, and the sample dissolved in *n*-hexane (1 mg/mL) was injected to the column (5 μL).

For the HPLC analysis of $\mathbf{P_{97}}$, $\mathbf{P_{70}}$, and their mixture ($\mathbf{P_{90}}$/$\mathbf{P_{70}}$) on Column **3**, a gradient elution using *n*-hexane and chloroform was employed. Since P3HT is insoluble in hexane, we used a chloroform/hexane mixture at a 7:3 (v/v) ratio to inject the sample. After sample injection, the eluent composition was gradually shifted to a hexane-rich mixture to facilitate effective separation. The gradient program was as follows: %chloroform = 70% (0.5 CV) | 70%-(2.5 CV)-100% (flow rate: 0.1 mL/min). The column temperature was 30 °C, and the sample dissolved in mixed solvent of *n*-hexane and chloroform (0.08 mg/mL) (*n*-hexane/chloroform = 30/70, v/v) was injected to the column (10 μL).

5. Batch Adsorption and Extraction Procedures

The solvent-assisted insertion of P3HT into **3** and the subsequent polymer isolation steps are described. These steps together enable RR-based polymer extraction on a batch scale.

Solvent-assisted P3HT insertion into 3. P_{90} (40 mg) was dissolved in 3 mL of chloroform, followed by the addition of **3** (120 mg). The mixture was left to stand at 25 °C (or 70 °C) in open air until the solvent gradually evaporated. The resulting solid was further dried under reduced pressure at 100 °C for 3 h. To remove unadsorbed P3HT remaining on the MOF surface, the solid was dispersed in a 7:3 (v/v) *n*-hexane/chloroform mixture and recovered by centrifugation. This washing process was repeated ten times to ensure complete removal of uninserted P3HT. Finally, the solid was dried under reduced pressure at 100 °C for 3 h, yielding the inclusion composite, **3**⊃P3HT (89 mg).

Isolation of P3HT from the composite. 3⊃P3HT composite (10 mg) was added to a 0.05 M water-methanol (1/1, v/v) solution of ethylenediaminetetraacetic acid tetrasodium salt (EDTA·4Na). The mixture was stirred at 25 °C for 1 h to digest the host **3** framework. Chloroform (5 mL) was then added, and the organic phase was extracted and dried over anhydrous Na_2SO_4. The solvent was removed under reduced pressure, yielding the isolated P3HT previously adsorbed in **3** (0.26 mg).

6. Flash Chromatography on a Preparative MOF Column

The preparative **3**-packed column for medium-pressure flash chromatography (20 mm I.D. × 150 mm L.) was prepared using a tapping method adapted from the literature.[28] Specifically, 13 g of dried **3** was placed into a glass column (20 mm I.D. × 150 mm L.) and packed by tapping, resulting in the column for preparative separation. The packed column was equilibrated with *n*-hexane before use. Subsequently, 20 mg of the crude material (**P_{90}**) was loaded onto the column and the fractionation was performed using the binary gradient program. Since **P_{90}** is insoluble in hexane, we used a chloroform/hexane mixture at a 7:3 (v/v) ratio to load the sample onto the MOF column. After loading, the eluent composition was gradually shifted to a hexane-rich mixture to facilitate effective separation. The gradient program was as follows: %chloroform = 30% (0.8 CV) | 30%-(1.33 CV)-70% (flow rate: 5 mL/min). The sample elution was monitored with UV detector operating at 400 nm. The sample was collected at different CVs and evaporated to dryness under reduced pressure. The resultant P3HT in each fraction was dissolved in $CHCD_3$ and analyzed by ^1H NMR.

7. Supporting Tables

Table S1. Synthetic conditions and properties of P3HT samples used in this study.

Code	Polymerization method	Catalyst Concentration (mM)	Monomer concentration (M)	M_n (g/mol)[a]	$Đ$[a]	RR[b]
$\mathbf{P_{90}}$	GRIM	$Ni(dppp)Cl_2$ 0.74	0.094	15,700	1.36	90.2%
$\mathbf{P_{94}}$	GRIM	$Ni(dppp)Cl_2$ 1.5	0.19	22,400	1.41	93.7%
$\mathbf{P_{97}}$	GRIM	$Ni(dppp)Cl_2$ 1.6	0.20	21,200	1.07	96.6%
$\mathbf{P_{77}}$	Oxidative	$FeCl_3$ 8.0×10^2	0.20	21,000[c]	1.08[c]	76.8%

[a]Determined by analytical SEC in THF calibrated with polystyrene standards.
[b]Determined by ^{1}H NMR.
[c]Fractionated by preparative recycling SEC.

Table S2. Results of the batch insertion experiments.

Entry	Temperature used for the insertion step	RR of original P3HT	RR of P3HT adsorbed in **3**	Amount of P3HT adsorbed in **3**
1	25 °C	90.2% ($\mathbf{P_{90}}$)	94.2%	12 mg/g
2	25 °C	93.7% ($\mathbf{P_{94}}$)	97.5%	38 mg/g
3	70 °C	90.2% ($\mathbf{P_{90}}$)	92.1%	90 mg/g
4	70 °C	93.7% ($\mathbf{P_{94}}$)	93.7%	225 mg/g

8. Supporting Figures

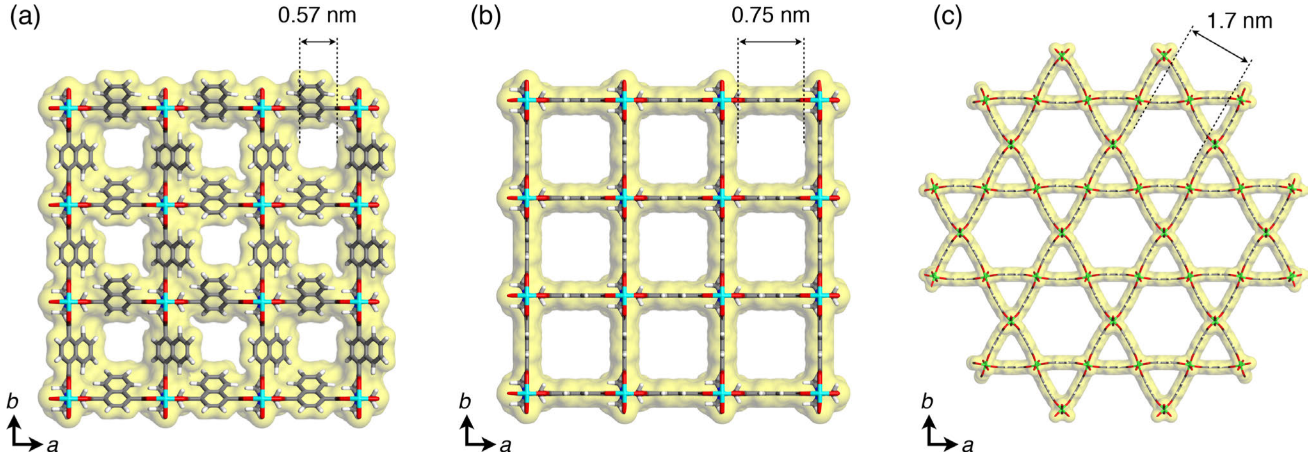

Figure S1. Framework structures overlayed with solvent-accessible surfaces of (a) $[Zn_2(ndc)_2(ted)]_n$ (**1**), (b) $[Zn_2(ndc)_2(ted)]_n$ (**2**), and $[In(bdc)(OH)]_n$ (**3**), viewed from the *c*-axis. Color code: gray, C; red, O; aqua, Zn; green, In. The effective pore diameter of **1**, **2**, and **3** along the *c*-axis measuring 0.57 nm, 0.75 nm, and 1.7 nm, respectively.

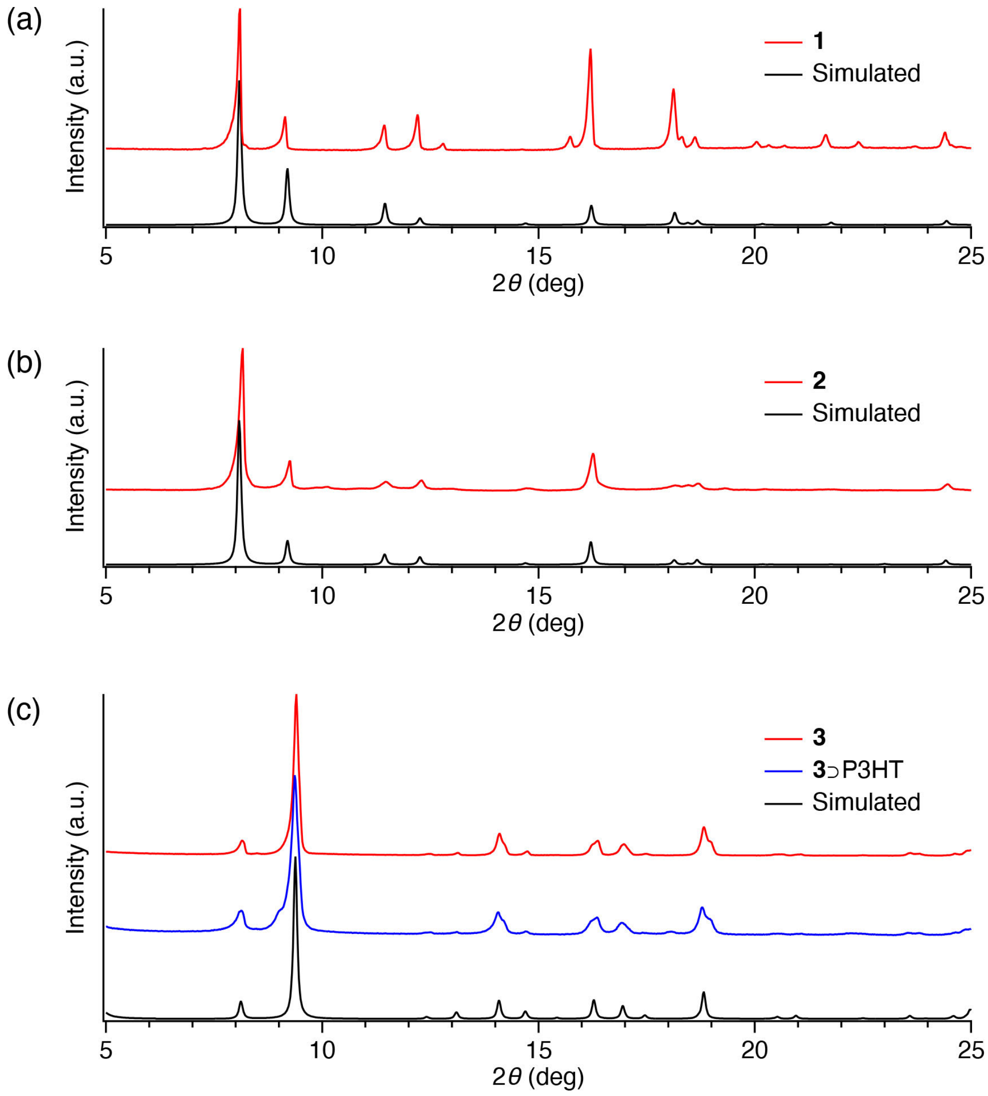

Figure S2. PXRD patterns of (a) **1**, (b) **2**, and (c) **3**. Red lines indicate experimental data. Black lines represent simulated patterns generated from crystallographic data for each MOF. In panel (c), the blue line shows the PXRD pattern of **3**⊃P3HT obtained after direct insertion of P3HT into **3**. The diffraction pattern of **3** remains unchanged, indicating that its crystal structure is preserved upon P3HT inclusion.

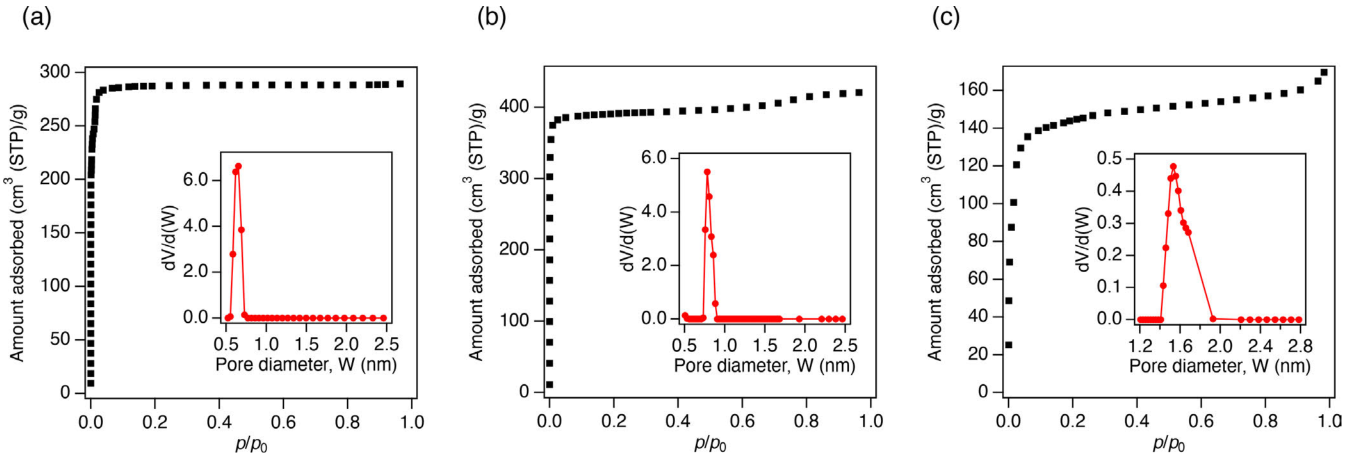

Figure S3. N_2 adsorption isotherms of (a) **1**, (b) **2**, and (c) **3** measured at 77 K. The inset shows the pore size distribution calculated using the Grand Canonical Monte Carlo (GCMC) method. The calculated pore sizes showed good agreement with the theoretical values derived from the single-crystal structure data of each MOF.

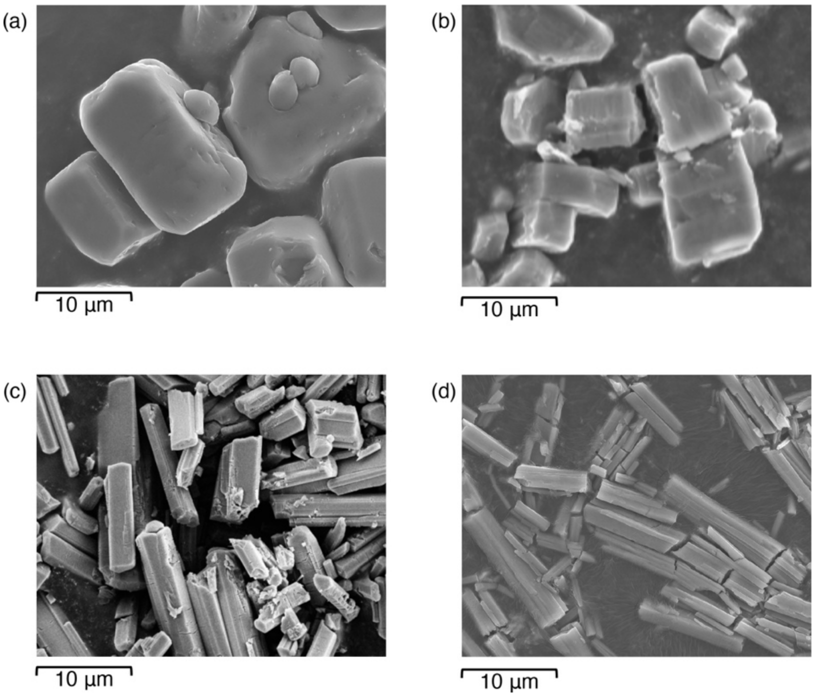

Figure S4. SEM images of (a) **1**, (b) **2**, and (c) **3** particles used for Columns **1**, **2**, and **3**, respectively. **3** was subsequently used for the direct P3HT insertion experiment, yielding (d) **3**⊃P3HT. The particle morphology of **3** was intact after the inclusion of P3HT.

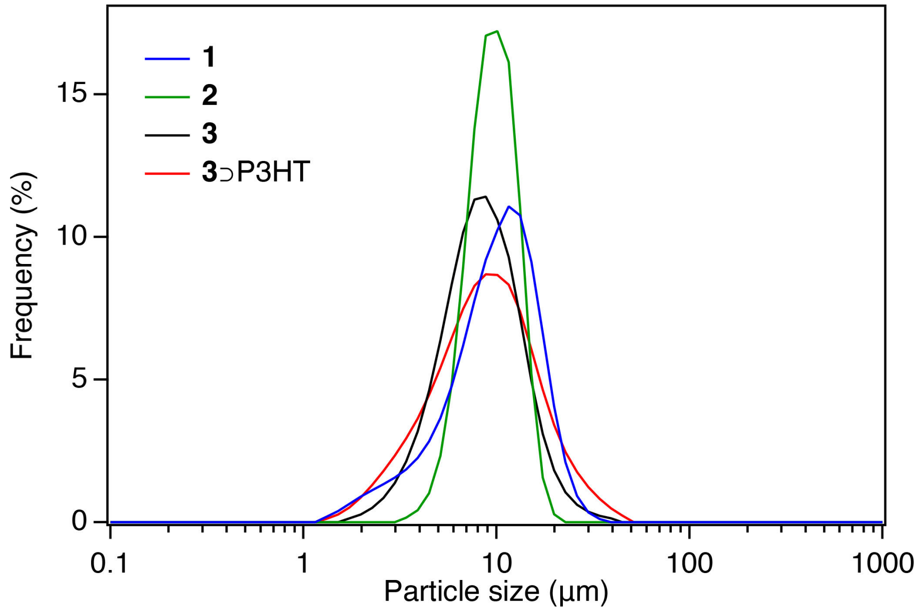

Figure S5. Particle size distribution data of **1** (blue), **2** (green), and **3** (black) particles used for Columns **1**, **2**, and **3**, respectively. The samples were dispersed in acetone and measured at 25 °C. **3** was subsequently used for the direct P3HT insertion experiment, yielding **3**⊃P3HT (red). The particle size of **3** remained unchanged after the inclusion of P3HT.

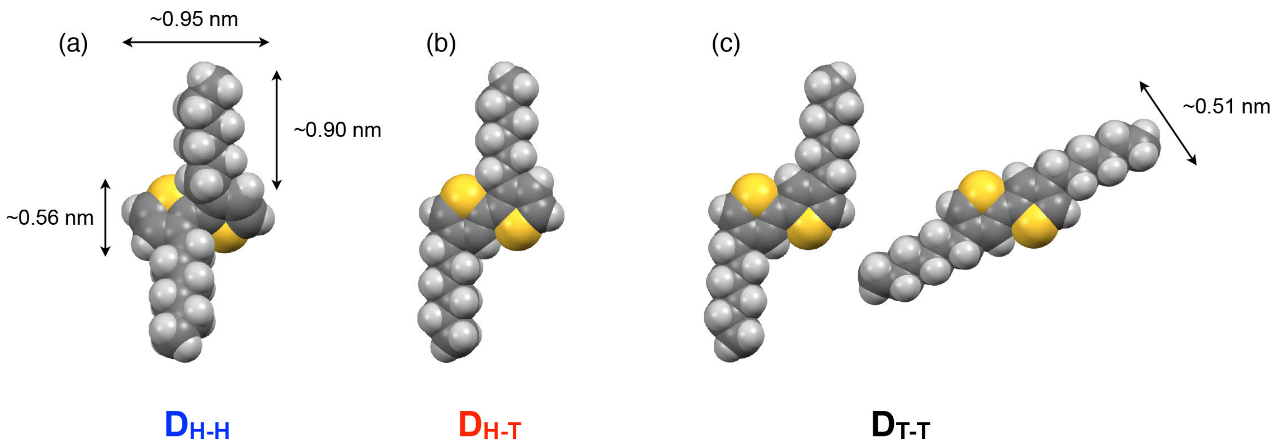

Figure S6. Molecular dimensions of (a) **$D_{H\text{-}H}$**, (b) **$D_{H\text{-}T}$**, and (c) **$D_{T\text{-}T}$**, modeled using molecular mechanics (MM) calculations. While **$D_{T\text{-}T}$** and **$D_{H\text{-}T}$** favor a coplanar structure, **$D_{H\text{-}H}$** adopts a twisted conformation due to steric hindrance of the side chains. As shown in panel (c), **$D_{T\text{-}T}$** can adopt a linear conformation by rotating its side chains, resulting in the thinnest structure among the three isomers.

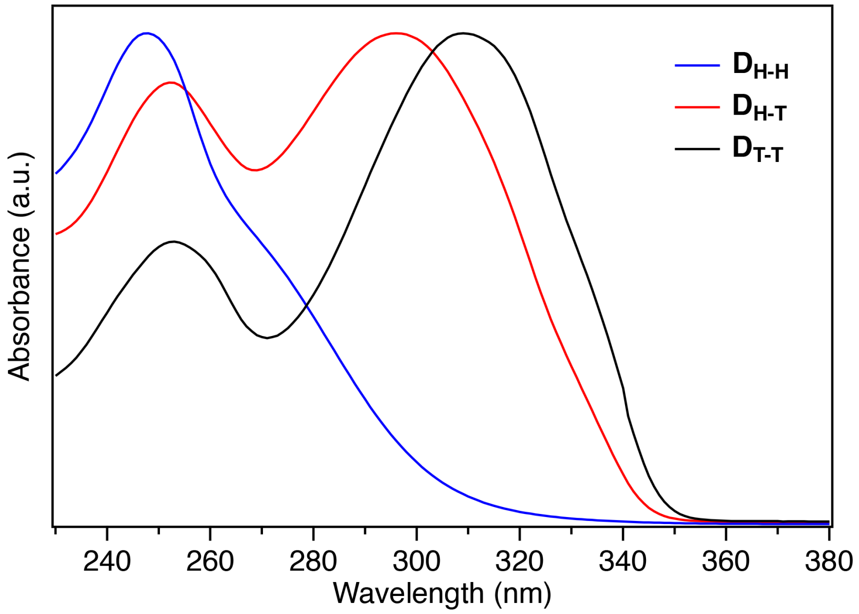

Figure S7. UV–vis absorption spectra of **D$_{H-H}$** (blue), **D$_{H-T}$** (red), and **D$_{T-T}$** (black), measured in *n*-hexane at 25 °C. **D$_{H-H}$** exhibited a significant blue shift in its maximum absorption wavelength compared to **D$_{H-T}$** and **D$_{T-T}$**. This trend is attributed to the molecular conformation of the dimeric isomers.[46] **D$_{H-H}$** adopts the *anti*-preferred conformation, caused by steric repulsion of the hexyl side chains, which leads to a shorter effective conjugation length. In contrast, **D$_{T-T}$** adopts a co-planar molecular conformation that extends the conjugation length, resulting in the longest maximum absorption wavelength among the three isomers.

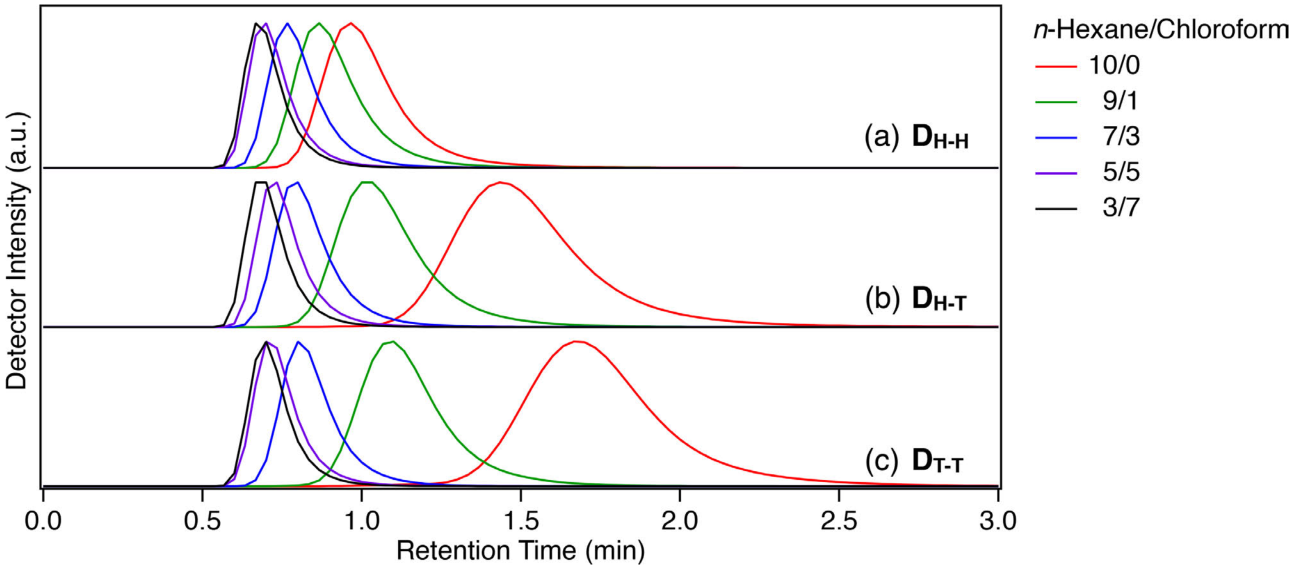

Figure S8. HPLC chromatograms of (a) $\mathbf{D_{H\text{-}H}}$, (b) $\mathbf{D_{H\text{-}T}}$, and (c) $\mathbf{D_{T\text{-}T}}$ obtained using Column **3** with mixed *n*-hexane/chloroform eluents at 40 °C and a flow rate of 1 mL/min. Solvent compositions of *n*-hexane/chloroform were 10/0 (red), 9/1 (green), 7/3 (blue), 5/5 (purple), and 3/7 (black). Increasing the concentration of chloroform reduces retention, enabling systematic modulation of column affinity toward the oligothiophenes.

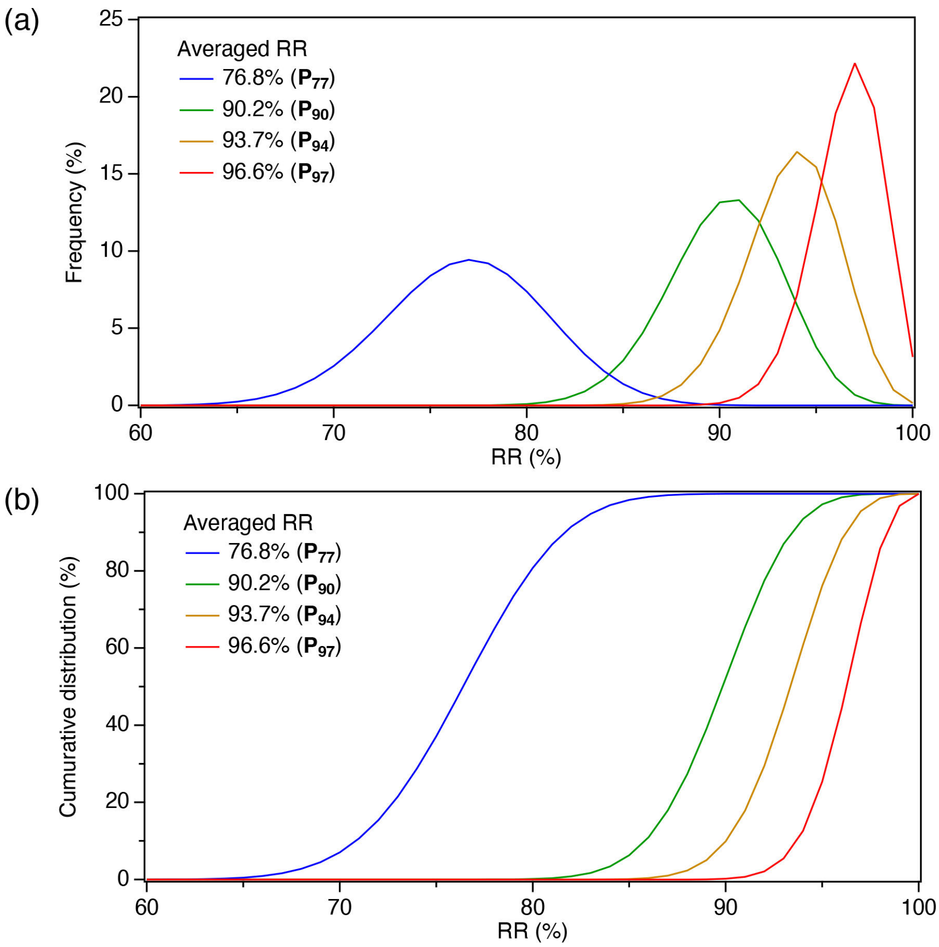

Figure S9. (a) Simulated binomial distribution and (b) its cumulative profiles, showing the probability of individual P3HT chains possessing specific RR values in mixtures generated by a statistical monomer coupling process. The profiles correspond to average RR values of 76.8% (blue), 90.2% (green), 93.7% (orange), and 96.6% (red), representing the synthesized P3HT samples $\mathbf{P}_{77}$, $\mathbf{P}_{90}$, $\mathbf{P}_{94}$, and $\mathbf{P}_{97}$, respectively. Specifically, for example, the fraction of high-RR chains exceeding 95% RR is 0.00008% in $\mathbf{P}_{77}$, 6.5% in $\mathbf{P}_{90}$, 39% in $\mathbf{P}_{94}$, and 87% in $\mathbf{P}_{97}$.

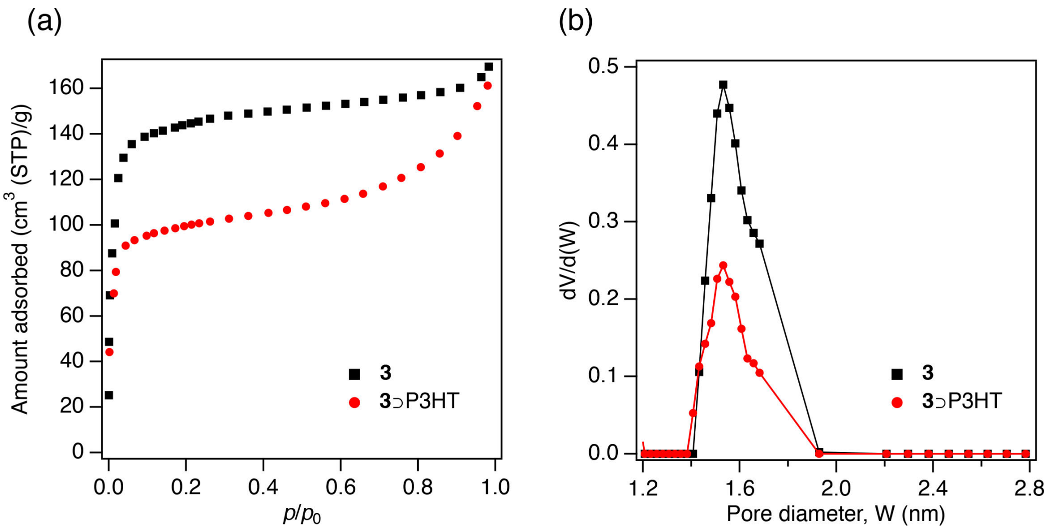

Figure S10. (a) N_2 adsorption isotherms and (b) pore size distributions calculated using the GCMC method for **3** (black square) and **3**⊃P3HT (red circle), measured at 77 K.

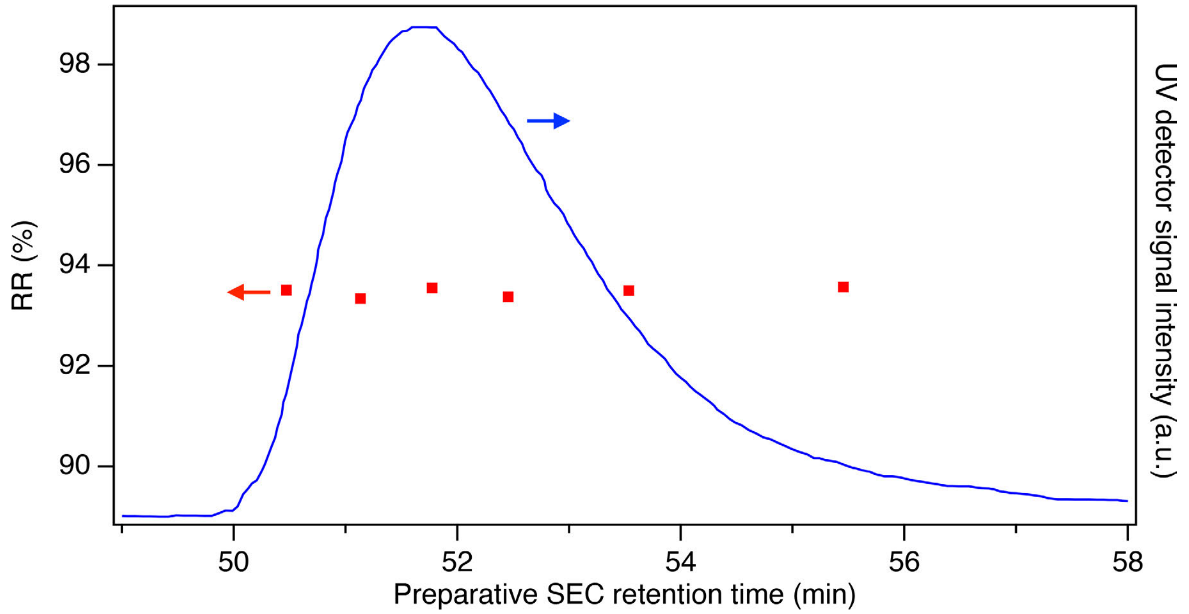

Figure S11. Preparative SEC trace of **P_{94}** (blue line, M_n = 22,400, $Đ$ = 1.41) and the RR values of different molecular weight fractions collected at the given retention time frame (red squares). The RR values remained consistent across all fractions, indicating that the regioregularity of P3HT is inherently independent of its molecular weight.

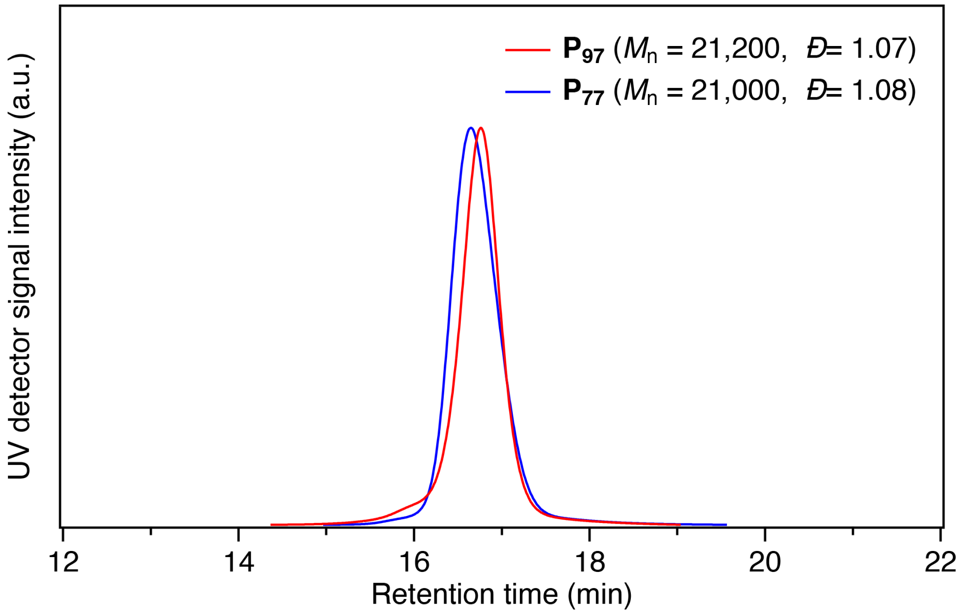

Figure S12. SEC traces of P97 (red) and P77 (blue) samples, recorded using THF as the eluent at 40 °C (flow rate: 1 mL/min). The molecular weights and distribution were obtained relative to polystyrene standards.

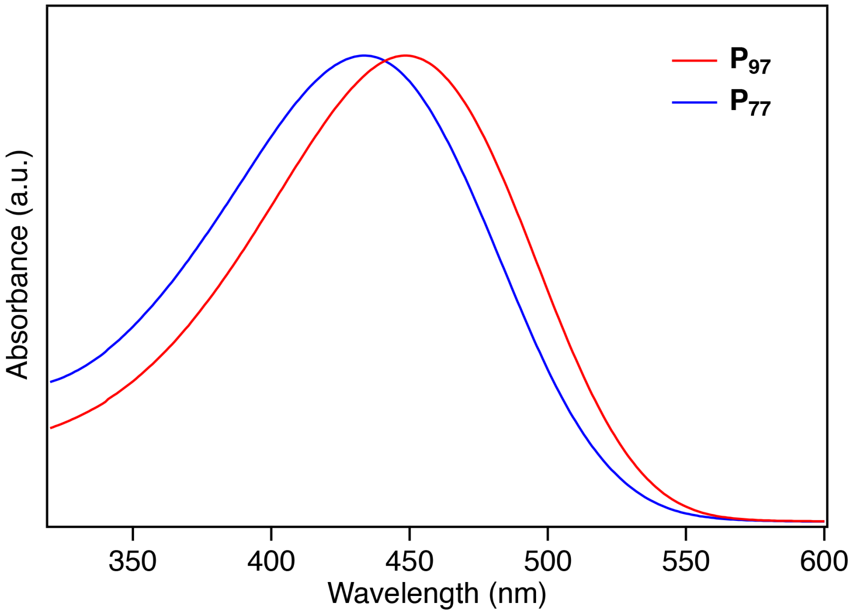

Figure S13. UV-vis spectra of **P₉₇** (red) and **P₇₇** (blue), recorded in chloroform (10 µg/mL) at 25 °C. Maximum absorption wavelengths (λ_{max}) were 449 nm and 434 nm, respectively.

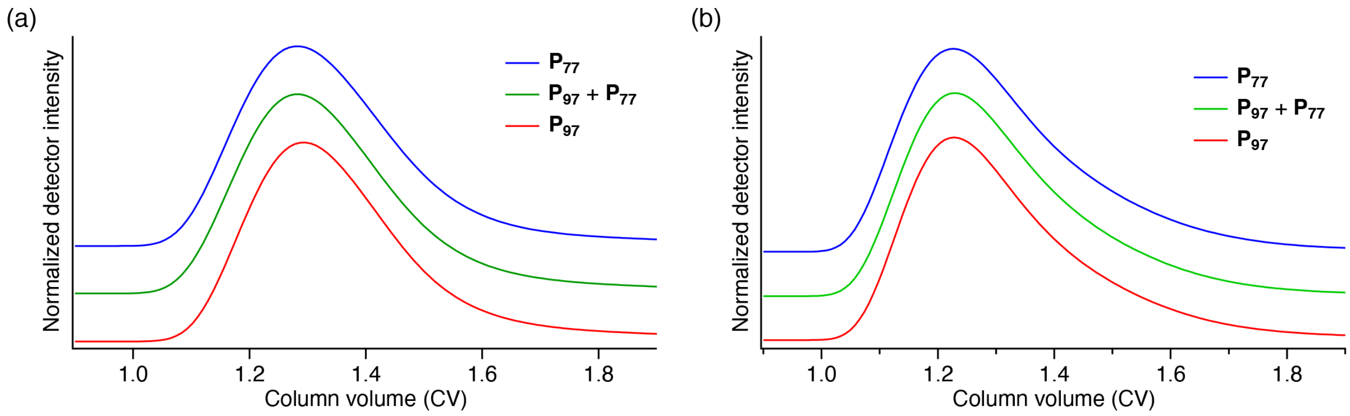

Figure S14. HPLC chromatograms recorded on (a) Column **1** and (b) Column **2** for $\mathbf{P_{77}}$ (blue), $\mathbf{P_{97}}$ (red), and the mixture of $\mathbf{P_{97}}$/$\mathbf{P_{77}}$ (1/1, w/w) (gradient: hexane/chloroform = 30/70 (0.5 CV) | 30/70-(2.5 CV)-0/100; temperature: 30 °C; flow rate: 0.1 mL/min; monitoring wavelength = 433 nm). Columns **1** and **2** showed no RR-based separation for P3HT.

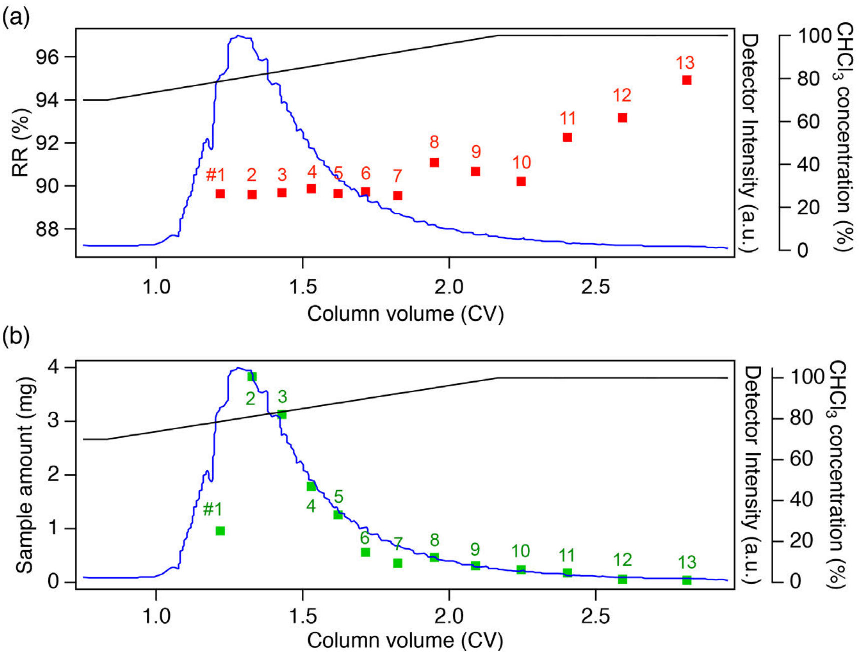

(c)

#Fraction	RR (%)	Sample amount (mg)
1	89.6	0.96
2	89.6	3.83
3	89.7	3.13
4	89.9	1.79
5	89.6	1.26
6	89.7	0.56
7	89.5	0.36
8	91.1	0.47
9	90.7	0.31
10	90.2	0.24
11	92.3	0.18
12	93.2	0.06
13	94.9	0.04
Total recovery		13.2

Figure S15. The chromatogram of the flash chromatography of $\mathbf{P_{90}}$ using the preparative column packed with **3** (Figure 5b), showing (a) the RR value and (b) the amount of each collected fraction as a function of retention time. Fractions are numbered from #1 to #13. (c) A table listing the RR values and corresponding sample amounts for each fraction. The initial sample loading was 20 mg, and the total amount recovered was 13.5 mg, corresponding to a recovery rate of 66%.

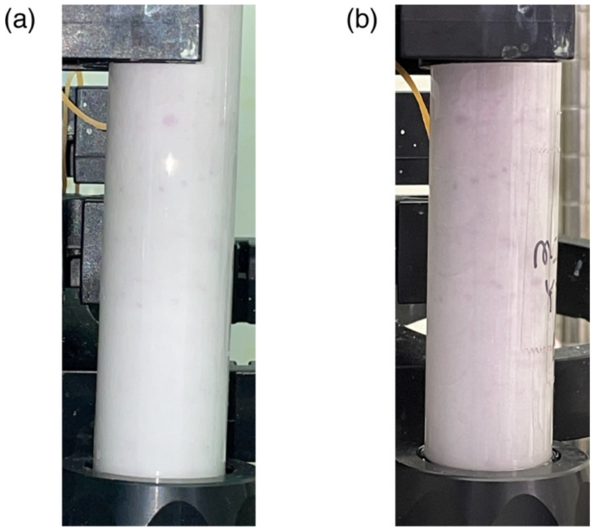

Figure S16. Photographs of the preparative column packed with **3**. (a) before use and (b) after performing the separation of $\mathbf{P_{90}}$. A noticeable color change from white to light purple was observed after use.

9. Supporting References

[44] S. S. Roy, S. K. Patra, *Eur. J. Inorg. Chem.* **2019**, 2193–2201.

[45] J.-C. Li, S.-H. Lee, Y.-B. Hahn, K.-J. Kim, K. Zong, Y.-S. Lee, *Synth. Met.* **2008**, *158*, 150–156.

[46] M. Breza, V. Lukeš, I. Vrábel, J. Mol. Struct.: *THEOCHEM* **2001**, *572*, 151–160.
